# Supplementary material for: Structure-specific rigid dose accumulation dosimetric analysis of ablative stereotactic MRI-guided adaptive radiation therapy in ultracentral lung lesions
Source: Commun Med (Lond). 2024 May 22;4:96. doi: 10.1038/s43856-024-00526-7 (PMC11111790; doi:10.1038/s43856-024-00526-7)
Supplement: Supplementary file 2 — Supplementary Information [file 43856_2024_526_MOESM2_ESM.pdf]

Structure-Specific Rigid Dose Accumulation Dosimetric Analysis of Ablative Stereotactic MRI-Guided Adaptive Radiation Therapy in Ultracentral Lung Lesions

J.M. Bryant[1]; Ruben Cruz-Chamorro[1]; Alberic Gan[2]; Casey Liveringhouse[1]; Joseph Weygand[1]; Ann Nguyen[2]; Emily Keit[1]; Maria L. Sandoval[1]; Austin J. Sim[3]; Bradford A. Perez[1], Thomas J. Dilling[1]; Gage Redler[1]; Jacqueline Andreozzi[1]; Luis Nardella[1]; Arash O. Naghavi[1]; Vladimir Feygelman[1]; Kujtim Latifi[1]; Stephen A. Rosenberg[1]

[1]Department of Radiation Oncology; H. Lee Moffitt Cancer Center & Research Institute; Tampa FL
[2]University of South Florida Health Morsani College of Medicine; Tampa FL
[3]Department of Radiation Oncology; James Cancer Hospital, Ohio State University Comprehensive Cancer Center; Columbus OH

Supplementary Table 1. Prescription coverage goals, dose constraints, and adaptation workflow trigger parameters

**Prescription coverage goals**				
**Target Volume**	**Dose (Gy)**	**Dose (Gy) per fx**	**Max dose (%)**	**Min dose (%)**
PTV*	60	7.5	120%	95%
GTV	60	7.5	120%	

**Dose constraints**	
**Organ at risk**	**Objective**
Lung	V20 Gy < 5%<br>V15 Gy < 10%<br>D_{Mean} < 6 Gy
Ipsilateral lung - PTV	V5 Gy < 35%
Lung total	1000 cc < 13 Gy<br>1500 cc < 12 Gy
Contralateral lung	D_{Mean} < 1.7 Gy<br>V5 Gy < 10%
Spinal cord	D_{Max} ≤ 15 Gy
Heart/Pericardium	D_{Max} < 44 Gy<br>V32 Gy < 15 cc
Chest wall	V30 Gy < 30 cc
Skin	D_{Max} < 39.1 Gy
**Critical constraints triggering online adaptation**	
**ROI**	**Trigger parameter**
Bronchial tree	D_{Max} ≤ 62 Gy
Esophagus	D_{Max} ≤ 40 Gy<br>V27.5 Gy ≤ 5 cc
Aorta, Vena Cava, or Azygous vein	D_{Max} ≤ 63 Gy<br>V56 Gy ≤ 10 cc

Abbreviations. GTV: gross tumor volume; PTV: planning target volume; ROI: region of interest
*PTV = GTV + 3 mm
Dose constraints of a particular volume represented with Volume (V) dose (in Gy) followed by the volume in relation to this dose (e.g., Lung V20 Gy < 5% means limiting 20 Gy to less than 5% of the total lung volume). Volume constraints of a particular dose is represented with Dose (D) volume followed by the dose in relation to this volume (e.g., Bronchial tree D_{Max} ≤ 62 Gy means limiting the maximum point dose within the bronchial tree to ≤ 62 Gy)

Table that summarizes the parameters used for treatment planning and online adaptation triggers for ultracentral lung tumors.

Supplementary table 2. Base, delivered, and predicted plans per fraction dosimetry parameters

ROI and Dosimetric Parameters	Median (IQR)		
	Base (n = 14)	Delivered (n = 112)	Predicted (n = 109)
Proximal bronchial tree			
Max (Gy)	61.9 (60.0 – 62.9)	61.7 (59.6 – 62.9)	61.0 (57.3 – 63.1)
0.03cc (Gy)	60.7 (59.5 – 61.3)	60.7 (57.1 – 61.7)	59.8 (55.7 – 62.2)
0.1cc (Gy)	58.4 (56.3 – 59.8)	58.0 (53.9 – 60.4)	58.0 (51.5 – 605.)
Target coverage			
GTV			
60Gy (%)	95.8 (90.3 – 99.7)	96.3 (92.0 – 99.6)	96.4 (91.1 – 99.2)
57Gy (%)	99.7 (96.7 – 100)	99.8 (97.4 – 100)	99.5 (96.1 – 100)
70Gy (%)	6.9 (0.5 – 28.7)	3.8 (0.3 – 30.6)	23.5 (3.3 – 35.0)
Min (Gy)	55.7 (41.6 – 59.1)	54.4 (43.9 – 58.8)	53.2 (39.3 – 58.5)
Max (Gy)	72.7 (70.8 – 76.2)	72.6 (70.7 – 78.3)	77.8 (73.4 – 80.9)
0.03cc (Gy)	72.0 (70.4 – 75.7)	72.1 (70.4 – 77.8)	77.2 (72.3 – 80.1)
0.1cc (Gy)	71.8 (70.0 – 74.8)	71. 5 (69.7 – 77.3)	76.0 (71.5 – 78.9)
PTV			
57Gy (%)	97.0 (89.8 – 99.6)	97.5 (90.5 – 99.5)	95.1 (88.9 – 98.5)
Min (Gy)	44.7 (28.8 – 55.7)	61.7 (59.6 – 62.9)	41.3 (27.0 – 53.4)
Abbreviations. GTV: gross tumor volume; IQR: interquartile range; PTV: planning target volume; ROI: region of interest			

Table that summarizes the median dosimetric parameters for the proximal bronchial tree and target coverage for the base, delivered, and predicted plans.
