# Supplementary material for: Structure-specific rigid dose accumulation dosimetric analysis of ablative stereotactic MRI-guided adaptive radiation therapy in ultracentral lung lesions
Source: Commun Med (Lond). 2024 May 22;4:96. doi: 10.1038/s43856-024-00526-7 (PMC11111790; doi:10.1038/s43856-024-00526-7)
Supplement: Supplementary file 3 — Description of Additional Supplementary Files [file 43856_2024_526_MOESM3_ESM.pdf]

Description of Additional Supplementary Files

File name-Supplementary Data 1

File description-Data used to generated figures within the article.
