# Supplementary material for: Structure-specific rigid dose accumulation dosimetric analysis of ablative stereotactic MRI-guided adaptive radiation therapy in ultracentral lung lesions
Source: Commun Med (Lond). 2024 May 22;4:96. doi: 10.1038/s43856-024-00526-7 (PMC11111790; doi:10.1038/s43856-024-00526-7)
Supplement: Supplementary file 5 — Reporting Summary [file 43856_2024_526_MOESM5_ESM.pdf]

nature portfolio

Corresponding author(s): John Michael Bryant and Stephen A. Rosenberg

Last updated by author(s): 4/28/2024

Reporting Summary

Nature Portfolio wishes to improve the reproducibility of the work that we publish. This form provides structure for consistency and transparency in reporting. For further information on Nature Portfolio policies, see our Editorial Policies and the Editorial Policy Checklist.

Statistics

For all statistical analyses, confirm that the following items are present in the figure legend, table legend, main text, or Methods section.

n/a	Confirmed	
☐	☒	The exact sample size (*n*) for each experimental group/condition, given as a discrete number and unit of measurement
☒	☐	A statement on whether measurements were taken from distinct samples or whether the same sample was measured repeatedly
☐	☒	The statistical test(s) used AND whether they are one- or two-sided<br>*Only common tests should be described solely by name; describe more complex techniques in the Methods section.*
☒	☐	A description of all covariates tested
☒	☐	A description of any assumptions or corrections, such as tests of normality and adjustment for multiple comparisons
☐	☒	A full description of the statistical parameters including central tendency (e.g. means) or other basic estimates (e.g. regression coefficient) AND variation (e.g. standard deviation) or associated estimates of uncertainty (e.g. confidence intervals)
☐	☒	For null hypothesis testing, the test statistic (e.g. F, t, r) with confidence intervals, effect sizes, degrees of freedom and P value noted<br>*Give P values as exact values whenever suitable.*
☒	☐	For Bayesian analysis, information on the choice of priors and Markov chain Monte Carlo settings
☐	☒	For hierarchical and complex designs, identification of the appropriate level for tests and full reporting of outcomes
☒	☐	Estimates of effect sizes (e.g. Cohen's *d*, Pearson's *r*), indicating how they were calculated

Our web collection on statistics for biologists contains articles on many of the points above.

Software and code

Policy information about availability of computer code

Data collection	Data for cumulative plans were generated and collected from Mirada image management system (Mirada Medical Ltd., Oxford, UK). Data for individual plans were collected from the MRIdian treatment planning system (ViewRay Inc., Mountain View, CA) .
Data analysis	All statistical tests were performed with SPSS version 29 software (IBM, Armonk, New York, United States).

For manuscripts utilizing custom algorithms or software that are central to the research but not yet described in published literature, software must be made available to editors and reviewers. We strongly encourage code deposition in a community repository (e.g. GitHub). See the Nature Portfolio guidelines for submitting code & software for further information.

Data

Policy information about availability of data

All manuscripts must include a data availability statement. This statement should provide the following information, where applicable:

- Accession codes, unique identifiers, or web links for publicly available datasets
- A description of any restrictions on data availability
- For clinical datasets or third party data, please ensure that the statement adheres to our policy

De-identified minimum dataset available upon request to corresponding authors. DICOMs are not available to protect patient privacy.

nature portfolio | reporting summary

March 2021

Human research participants

Policy information about studies involving human research participants and Sex and Gender in Research.

Reporting on sex and gender	Sex data collected for all patients but none used during analysis. Sex data reported as part of relevant immutable baseline patient characteristic profile, as is standard within clinical oncological reporting. Gender identity data were not collected.
Population characteristics	Age, sex, performance status, cancer histology, cancer site, cancer T stage, cancer lesion maximum diameter, pulmonary lobe location, lesion ultracentral status, lesion distance from proximal bronchial tree, tumor volume, target volume, systemic therapy details, and prior thoracic RT details were collected as part of population characteristics.
Recruitment	Retrospective study
Ethics oversight	Our study was approved by our institutional review board (full details redacted to protect authors' anonymity during review process, available upon request)

Note that full information on the approval of the study protocol must also be provided in the manuscript.

Field-specific reporting

Please select the one below that is the best fit for your research. If you are not sure, read the appropriate sections before making your selection.

☒ Life sciences ☐ Behavioural & social sciences ☐ Ecological, evolutionary & environmental sciences

For a reference copy of the document with all sections, see nature.com/documents/nr-reporting-summary-flat.pdf

Life sciences study design

All studies must disclose on these points even when the disclosure is negative.

Sample size	No sample size was pre-calculated. Our sample size of 14 patients were more than sufficient because they each generated a base plan, 8 predicted fraction plans, 8 delivered fraction plans, and four cumulative dose plans. These numbers are more than sufficient when analyzing using the non-parametric Wilcoxon Signed Rank test to compare matched samples (i.e., delivered versus predicted).
Data exclusions	No data were excluded
Replication	Full description of treatment planning, data collection, generation of rigid image registration to generate cumulative dose plans, statistical analyses, and definition are provided in-text
Randomization	This is a single cohort study that reports on all patients that met inclusion criteria. Clinical outcome analyses were descriptive in nature and dosimetry analyses were done between the paired samples of delivered and predicted plans for each patient; therefore, there was no role for randomization in this study.
Blinding	This is a single cohort study that reports on all patients that met inclusion criteria. Clinical outcome analyses were descriptive in nature and dosimetry analyses were done between the paired samples of delivered and predicted plans for each patient; therefore, there was no role for blinding in this study.

Reporting for specific materials, systems and methods

We require information from authors about some types of materials, experimental systems and methods used in many studies. Here, indicate whether each material, system or method listed is relevant to your study. If you are not sure if a list item applies to your research, read the appropriate section before selecting a response.

Materials & experimental systems

n/a	Involved in the study	
☒	☐	Antibodies
☒	☐	Eukaryotic cell lines
☒	☐	Palaeontology and archaeology
☒	☐	Animals and other organisms
☐	☒	Clinical data
☒	☐	Dual use research of concern

Methods

n/a	Involved in the study	
☒	☐	ChIP-seq
☒	☐	Flow cytometry
☒	☐	MRI-based neuroimaging

Clinical data

Policy information about clinical studies

All manuscripts should comply with the ICMJE guidelines for publication of clinical research and a completed CONSORT checklist must be included with all submissions.

Clinical trial registration	This is not a clinical trial. This is a retrospective study.
Study protocol	Full study details provided within methods section of manuscript with reference resource provided that contains additional details regarding adaptive MRI-guided radiation therapy used in this study.
Data collection	After obtaining institutional review board approval, 14 consecutive patients who received ablative SMART (i.e., BED10 > 100 Gy) for ultracentral lesions between October 2019 and January 2021 were identified. Ultracentral lesions were defined as per the Nordic-HILUS7 group A and B criteria: ultracentral group A lesions (i.e., gross tumor volume [GTV]) were defined as being ≤ 1 cm from the main bronchus and trachea and group B lesions were defined as being ≤ 1 cm from intermediate and lobar bronchi. Patients with primary lung cancers and metastatic lesions to the lung were included.
Outcomes	Toxicities were assessed using Common Terminology Criteria for Adverse Events (CTCAE) v.5.0. Acute toxicities were defined from the start of treatment to less than 90 days post-treatment. Late toxicities were defined as greater than or equal to 90 days from the end of treatment. Follow-up was defined from the end of SMART to the last contact or death. Clinical outcomes were estimated from end of SMART to last follow-up, progression, or death. Local control (LC) was defined as the absence of local failure (LF) as per Response Evaluation Criteria in Solid Tumors (RECIST) confirmed by CT, PET, or biopsy, marginal failure, or involved lobe failure, as per RTOG 08134. Overall survival (OS) was defined as freedom from death of any cause. Progression-free survival (PFS) was defined as the shortest interval of time to either local failure, distant failure, or death.
